# Progress of Research on Diffuse Axonal Injury after Traumatic Brain Injury

**DOI:** 10.1155/2016/9746313

**Published:** 2016-12-19

**Authors:** Junwei Ma, Kai Zhang, Zhimin Wang, Gang Chen

**Affiliations:** ^1^Department of Neurosurgery & Brain and Nerve Research Laboratory, The First Affiliated Hospital of Soochow University, 188 Shizi Street, Suzhou 215006, China; ^2^Department of Neurosurgery, Suzhou Kowloon Hospital Affiliated Shanghai Jiao Tong University, Suzhou 266021, China

## Abstract

The current work reviews the concept, pathological mechanism, and process of diagnosing of DAI. The pathological mechanism underlying DAI is complicated, including axonal breakage caused by axonal retraction balls, discontinued protein transport along the axonal axis, calcium influx, and calpain-mediated hydrolysis of structural protein, degradation of axonal cytoskeleton network, the changes of transport proteins such as amyloid precursor protein, and changes of glia cells. Based on the above pathological mechanism, the diagnosis of DAI is usually made using methods such as CT, traditional and new MRI, biochemical markers, and neuropsychological assessment. This review provides a basis in literature for further investigation and discusses the pathological mechanism. It may also facilitate improvement of the accuracy of diagnosis for DAI, which may come to play a critical role in breaking through the bottleneck of the clinical treatment of DAI and improving the survival and quality of life of patients through clear understanding of pathological mechanisms and accurate diagnosis.

## 1. Introduction

Diffuse axonal injury (DAI) is a brain injury characterized mainly as axonal injury of the white matter. It often follows brain trauma, which causes wide-ranging denaturation of white matter, focal hemorrhage, emergence of axonal retraction balls, and microglia clusters. DAI is often accompanied by other brain injuries, and this has caused patients severe brain damage or even placed them in a persistent vegetative state. According to reports made in recent years, the mortality rate of DAI is 42%–62% [1, 2]. DAI has been as an independent category of disease accepted by neurosurgery academic. However, there are currently no standard diagnostic criteria, and the relationship to other brain injuries needs to be investigated further in order to develop better clinical treatments for DAI. Below, the authors review the concept, pathological mechanism, and methods of clinical diagnosis of DAI.

## 2. Concept

DAI was formally named and accepted by the international academic community in 1982. It has gone through three conceptual stages in its history. The first period began in 1956, when Strich studied autopsies from 5 patients with severe closed brain trauma and proposed that degeneration of the diffuse white matter might be attributed to the physical damage to nerve fibers. The second period began in 1961, when this Strich studied 20 patients who had died of brain trauma. He found that the shearing force of the rotational acceleration of head movement (one of the main causes of brain injury) caused the nerve fibers to break and evoked diffuse degeneration of hemisphere and brainstem. This study provides a theoretical basis for future investigations of DAI. The third period began in the 1980s, when Adams and Gennerelli studied the mechanism of development and clinical pathology of DAI thoroughly and made prominent achievements, which were given great consideration when the international academic community selected a final name for this condition.

## 3. Pathological Mechanism of DAI

DAI usually presents a progressive course. It takes place after external injury involving shearing force, and it mainly manifests in the form of focal axonal changes and axonal breakage. And it can be divided into primary and secondary axonal injury. The pathological mechanism of DAI is very complicated, but a clear understanding of the pathological mechanism is very important to diagnosis, clinical treatment, and prognosis; pathological characterization has become a hot topic in neurosurgical research.

### 3.1. Pathological Mechanism of Primary Axonal Injury

#### 3.1.1. Formation of Axon Retraction Balls

The main cause of primary axonal injury was axonal breakage, retraction, and the formation of what is called axon retraction balls because of the shape of the swelling at the end of the axonal axis, which was caused by the external shear force and tension. The formation of these axon retraction balls was believed to lead to the final breakage of the axon. Currently, it is thought that the axon retraction balls cause axon breakage, so interrupting protein transport, and the individual axon retraction ball has been observed under microscopy at the end of broken axons. However, multiple recent studies have shown that the site of instant, strong shearing force or tension within the brain does not always match the site of actual injury. Animal studies have shown there to be no axon breakage immediately after brain trauma, and pathological examination suggested that the myelin of the axons had remained intact [3–6]. This has sparked debate over whether it is suitable to assess the number of injured axons by determining the total number of axon retraction balls after onset of DAI.

### 3.2. Pathological Mechanism of Secondary Axonal Injury

#### 3.2.1. Calcium Ion (Ca^2+^) Influx and Calcium-Protein-Mediated Structural Protein Hydrolysis and the Cytoskeleton Network of Degrading Axons

*(1) Ca* ^*2+*^ *Influx Activated the Signaling Pathway of Cysteine Protein.* After external instant shear force and tension act on the brain, the permeability of the axon membrane changes, and large amounts of Ca^2+^ enter the cells. The anterograde transport of axon plasma is gradually converted to retrograde transport, so activating the cysteine protein signal pathway and caspase-3. The inherent cellular calpain inhibitor calpastatin is hydrolyzed. A relatively high level of activated calpain accumulates within the cell, and this degrades the axonal cytoskeleton network. Recent studies have shown that influx of Ca^2+^ and degradation of the axonal cytoskeleton network are progressive events, during which axons usually maintain their morphology several hours after injury [7–10].

*(2) Calpain-Mediated Hydrolysis of Structural Protein.* Spectrin, also called cell ghost, is a structural protein found on the inner side of the membrane. It not only supports the lipid bilayer but also maintains the shape of red blood cells. It forms a transformable network beneath the plasma side of the membrane and so maintains the biconcave disk shape of red blood cells. During the early stage of injury, calpain-mediated hydrolysis of spectrin in focal axon was observed, as indicated by single and double markers' under immunohistological examination via light microscopy and electromicroscopy. Most axons show signs of calpain-mediated hydrolysis of spectrin 1-2 h after the injury. Related pathological changes include loss of microtubules, swelling of the mitochondria, and neurofilamentous knots, which indicate that calpain-mediated hydrolysis of structural protein and degradation of the cytoskeleton play important roles in the development and progression of DAI pathology [11–13].

#### 3.2.2. Mitochondrial Damage, Imbalance of Ion Homeostasis, Release of Proapoptotic Factors, and Activation of Caspase-Mediated Programmed Cell Death

Mitochondrial damage after onset of DAI mainly includes swelling and breakage of the mitochondrial crest and membrane. This type of focal damage of mitochondria closely related to Ca^2+^ influx. Ca^2+^ influx leads to changes in the permeability of the mitochondrial membrane and affects the opening of the switching pore in said membrane. The intake of small molecules causes the mitochondria to swell and break, which further not only disrupts the energy metabolism and ion homeostasis but also releases caspases and the activators of apoptosis, so triggering caspase-mediated progressive cell death. Caspases hydrolyze proteins severely in injured axons [14–17]. In this way, impairment of the mitochondria, imbalance in ion homeostasis, the release of proapoptotic factors, and activation of caspases are key contributors to the high mortality and poor prognosis of DAI.

#### 3.2.3. Changes in Transport Proteins, Such as Amyloid Precursor Protein (APP)

Amyloid precursor protein is a single transmembrane protein present in most cells and tissues. It has drawn a great deal of attention because it can be converted to toxic *β*-amyloid (A*β*) after protease hydrolysis. The use of immunohistology to assess changes in APP in axons is the gold standard of neuropathology and trauma model diagnosis of DAI [18, 19]. Once pathological changes take place, the anterograde transport of APP becomes disrupted, which causes focal aggregation of APP.

#### 3.2.4. Changes in Glia Cells

Increasing amounts of evidence show that changes in glia cells play very important roles in the development and progression of DAI. The morphological and functional changes in astrocytes, microglia, and oligodendrocytes that take place after onset of DAI and are called “glial reaction.” Glial cells become activated and involved in eliminating and engulfing particles expelled from the site of injury, extend projections to fill in cavities, form glial scars, and produce matrix metal proteins (MMPs) to reconstruct damaged extracellular matrices after the progression of DAI. Glia cells also express insulin-like growth factor-1, epithelial growth factor, and other neurotrophic growth factors in order to decrease the rate of neuronal death and neural injury after the progression of DAI [20, 21].

Astroglia (AS) is a major type of glial cells in the central nervous system (CNS) originating from neural ectoderm. The distribution of AS in the brain was regular (GFAP positive cells in hippocampus and dentate gyrus in obvious rules). This kind of order contributes to the position of the fixed relationship and the function of stable relationship between AS and neuron. And AS may also be involved in the complex functions of the brain activity, including learning and memory. When brain was injured, it usually leads to reactive hyperplasia of AS. Recently, it showed that AS clears hemorrhage in the early damage and degeneration necrosis tissue with macrophages and thereby promotes wound repair [22, 23]. Corresponding to different neurotransmitters and neuropeptide, there are many receptors in AS, such as 5-HT and *γ*-GABA. In recent years we thought that it (at least under the condition of in vitro) has almost all possible neurotransmitters functional receptors [24]. After being damaged, neurons produce more neurotransmitters than normal, so the receptors on the AS can upregulate and produce more growth factors to promote repairing of injury.

Oligodendrocyte (OLG) is myelin glial cells in the central nervous system and rich in grey and white matter of brain and spinal cord. The damage of OLG has far-reaching influence to white matter. Mechanical damage, ischemia, or axonal degeneration can cause the damage and apoptosis of OLG; otherwise, there is great relevance between axonal degeneration after brain injury and the apoptosis of OLG [25]. And the Fas and p75 receptor activation may be involved in apoptosis [26].

However, glial cells become activated further, to the point of overactivation, as DAI progresses. Overactivated glial cells continuously release inflammatory factors, such as IL-1*β* and TNF-*α*, and they release oxygen free radicals and cytotoxic substances, which elicits inflammatory responses, causes oxidative stress in brain tissue, and directly or indirectly induces neuronal death. Overactivation of glia cells causes the release of chondroitin sulfate proteoglycan, prevents the glia cells from reconstructing the extracellular matrix, inhibits axon growth, and weakens the ability of glial cells to eliminate products expelled from the site or injury. In this way, overactivated glial cells promote neuronal injury.

Activation of glia cells can also promote neuron-glia and glia-glia interactions. Previous studies have demonstrated that the chemokine CXCL-12, which is released from astrocytes, promotes the release of glutamate, which further promotes the release of large amounts of TNF-*α* from microglia. High concentrations of TNF-*α* impair the ability of microglia to eliminate glutamate, and this causes excitatory toxicity and injures neurons [13]. Astrocytes also release the anti-inflammatory factor IL-10, which inhibits the release of TGF-*β* from microglia and promotes the maturation of oligodendrocytes [27–30].

However, it remains unclear whether the activation of glial cells promotes injury or repair. The actual roles of the activation of glial cells require further investigation.

## 4. Diagnosis of DAI

### 4.1. Imaging Examination

#### 4.1.1. Computed Tomography (CT) and Traditional MRI Examination

CT allows rapid and reliable location of focal hemorrhages related to axonal injury, but it is difficult to find injuries other than hemorrhages, especially if they are small in size or involve needle-like bleeding.

Traditional MRI examination not only allows rapid location of hemorrhages, but it is also a sensitive and reliable way of locating nonhemorrhages. It has better resolution than CT scans and it is especially suitable for injuries to the posterior cranial fossa and deep white matter. However, it still has a high rate of false negative results for small lesions and mild DAI. Moreover, patients are often unable to complete the examination due to the long time requirements.

#### 4.1.2. Diffusion-Weighted MRI (DWI) and Diffusion Tension Imaging (DTI)

As medical science has progressed, more accurate methods of diagnosing DAI have been developed. Some of these are based on DWI and DTI. DWI involves using the anisotropy of protein to identify changes in white matter after onset of DAI. Studies have shown DWI to be an accurate method of examining nonhemorrhage injuries, especially at the sites within the cranial vault. However, this method is often not sufficiently accurate for the examination and diagnosis of injuries to the corpus callosum and grey matter. DTI, which was developed as an improved form of DWI, can be used to evaluate nerve alignment, injury context, and the microstructure of white matter effectively. It can also allow direct observation of the nerve alignment and the collection of abnormal morphology information regarding major nerve fibers. In this way, DTI can detect DAI in a highly sensitive way and allow estimation of the time elapsed from injury to examination.

#### 4.1.3. Gradient Echo Pulse Sequence-Susceptibility Weighted Imaging (GRE-SWI)

GRE-SWI can detect more minor hemorrhages and so indicate the severity of DAI more accurately than other methods can, which makes it especially suitable for early diagnosis of DAI.

GRE-SWI is different from proton density and T1 and T2 weighted imaging. This new imaging method is the use of magnetic susceptibility which is different between different organizations and imaging technology. And the key to imaging is magnetic sensitive material; in some tissues, such as venous blood, bleeding, and calcification, the magnetic susceptibility is different from that of surrounding tissues. On the one hand it can shorten T2^*∗*^; on the other hand, it can lead to blood vessels and surrounding tissues of different phase contrast.

Diffuse axonal injury (DAI) accounts for more than 30% of severe craniocerebral injury and is the main causes leading to a vegetative state or serious nerve dysfunction. Further clinical study found hemorrhage of DAI with worse prognosis than less bleeding. However, both CT and routine MRI are not sensitive to the smaller hemorrhage stove. GRE-SWI is very sensitive to hemoglobin metabolites, such as DNA, methemoglobin, hemoglobin, and hemosiderin. So, GRE-SWI can detect these metabolites more effectively than conventional MRI [31, 32]. So the GRE-SWI play an important role in the evaluating, treating of traumatic brain injury, and prognosis judging.

Although GRE-SWI is valuable for finding the minor hemorrhage in brain clinically, it still cannot make difference between other minor hemorrhages caused by patients related diseases, such as hypertension. And the acquisition and processing technology still needed further improvement, to improve the scanning speed, reduce artifacts, and improve the signal-to-noise ratio.

### 4.2. Neural Electrophysiology

Neural electrophysiology is one of noninvasive tools available for studying DAI. Animal studies have shown that rats with mild DAI have abnormal neural electrophysiology regardless of whether they have sustained any axonal injury [33]. Other studies have shown pathological changes and decreases in action potential in the axonal axis of the corpus callosum of mice with brain trauma. The action potential of both myelinated nerve fibers and unmyelinated nerve fibers in the corpus callosum has been found to decrease. Among those nerve fibers, myelinated fibers were found to recover their action potential gradually as their axons were repaired, while unmyelinated nerve fibers did not [34–38]. These findings indicated that the abnormal action potential of unmyelinated nerve fibers may play an important role in the disability associated with DAI.

### 4.3. Diagnosis Based on Biochemical Markers

Currently, commonly used biochemical markers for acute DAI diagnosis and analysis of the conditions and prognosis associated with DAI include *β*-APP, spectrin, and its decomposition products SBDP145 and SBDP150. Other markers include neurofilaments and the phosphorylated products of their tau subunits and hydrolyzation of myelin basic protein.

#### 4.3.1. *β*-APP

The detection of *β*-APP is currently considered the gold standard of DAI examination in forensic and laboratory settings. It is often used for early diagnosis of DAI.

Under normal conditions, the *β*-APP present in axons cannot be detected using immunohistochemistry. However, after onset of DAI, the disruption of transportation through the axoplasm causes *β*-APP to aggregate in the axons, bringing its concentration up to detectable level. This makes it suitable for use as a marker for early diagnosis of DAI. However, detection of *β*-APP by immunohistochemistry after onset of DAI can cause underestimation of the scope of axonal injury. Through more in-depth studies, detection of *β*-APP695, an isoform of *β*-APP, could provide more reliable and sensitive diagnosis of DAI [39]. Attention must be paid to diseases that can cause clinically abnormal axonal metabolism, in which *β*-APP has been shown present via immunohistochemistry. In this way, patients' disease history must be taken into consideration, which would increase the accuracy of diagnosis via immunohistochemical examination of *β*-APP.

#### 4.3.2. Spectrin-II Subunit

The spectrin-II subunit is present within the neuron body, dendrite, and axons. Along with neurofilaments and microtubule-associated proteins, it plays an important role in maintaining neuron morphology and function. The spectrin-II subunit of calpain degradation products (SBDP) detected in cerebral cortex, cortex medullary junction, corpus callosum, and cerebrospinal fluid following DAI mainly include SBDP-150 and SBDP-120. The trends in the changes of the concentrations of SBDP-150 and SBDP-120 in the cerebral cortex and corpus callosum have been shown to be similar [40], which indicate that, after onset of DAI, calpain-induced necrosis is an important pathological mechanism of DAI. However, the trends in the concentrations of SBDPs in cerebrospinal fluid are not synchronous with those of the brain, and the trends in the concentrations of degradation products from different subunits of spectrin are also different. One possible reason for this is that the proteins released from the brain parenchyma must be transported into the cerebrospinal fluid via the intercellular fluid, while proteins released from injured neurons in the subarachnoid space can be released directly into the brain [41]. In this way, the measurement of the expression of different subunits of spectrin expression could be used to assess the severity of DAI, show whether it is associated with focal or diffuse functional impairment, and provide some basis for predicting the pathological mechanism of DAI.

#### 4.3.3. Neurofilaments

Neurofilaments are involved in the cytoskeleton and play an important role in axonal transportation. Neurofilaments are composed mainly of light chains (NF-L), medium chains (NF-M), and heavy chains (NF-H). After onset of DAI, the spatial configurations of NF-L, NF-M, and NF-H peptides were different, according to the severity of DAI. In mild and moderate DAI, three types of NF subunits presented focal disorder. In moderate DAI, compact area shows up in NF. The axons and microtubule protein decreased significantly. Phosphorylated neurofilament was hydrolyzed and finally resulted in neurofilament collapse. Because NF-H can be detected in serum after onset of DAI and increased from 6 h, peaked at 12 h and 48 h, and decreased to normal level on day 7 [42, 43]. NF-H is considered the most convenient marker of DAI diagnosis. NF-L is the most sensitive and specific marker of DAI diagnosis. NF-M must be investigated further if it can be used as a specific marker of DAI diagnosis.

#### 4.3.4. Tau Protein

Tau is the most abundant protein in microtubule-related proteins. Tau contains a phosphoric acid group. Each molecule of tau contains 2-3 phosphoric acid groups. Overphosphorylated tau groups lose their normal transport function in axons and in turn inhibit the assembly and promote dissemble of microtubule, finally causing axonal breakage. After onset of DAI, tau was depolymerized to C-tau by calpain, which can be detected in large amounts in cerebrospinal fluid. The detection level of C-tau in the cerebrospinal fluid is negatively correlated to the severity of DAI of patients in clinical settings [44, 45]. In this way, the detection of C-tau in cerebrospinal fluid was used to quantitatively evaluate the severity of axonal injury. Investigation has shown that once the C-tau level in patients' cerebrospinal fluid reaches 2.126 mg/mL, the accuracy of prognosis of the mortality rate reaches 100% and specificity rises above 80% [46]. However, C-tau detected in serum was not found to facilitate effective evaluation of prognosis. For this reason, the detection of C-tau levels in the cerebrospinal fluid is considered one of the most suitable biochemical markers for clinical diagnosis of DAI.

#### 4.3.5. Myelin Basic Protein (MBP)

Myelin basic protein (MBP) is the main protein in myelin in the central nervous system (CNS). It is present on the plasma side of myelin, where it keeps the protein's structure and function stable. It is specific to nerve tissue. Because of the blood-brain barrier (BBB), MBP is readily released into cerebrospinal fluid, and a very small amount of MBP is released into the blood. After onset of DAI, the CNS is damaged and the BBB can be completely destroyed. The changes in the permeability of BBB result in the increase of MBP levels in serum [47]. Determination of MBP level in serum can indicate its quantity in a timely manner, and the samples for determination are easy to collect. Scholars both within and outside of China have reported that MBP could be a suitable index of the severity of CNS injury [48]. In the same way, the determination of MBP levels in serum and cerebrospinal fluid could facilitate preliminary judgement of the severity of DAI and allow objective evaluation of the progression and prognosis of DAI. However, the sensitivity of the detection of serum MBP is not currently ideal and the use of MBP detection in clinical settings is limited.

#### 4.3.6. Others

Other biomarkers for diagnosis of DAI include cyclooxygenase-2, aquaporin-4, inflammatory reaction factors (such as IL-1*β*, IL-6, and TNF), and basic fibroblast growth factor. These factors can facilitate diagnosis of continued injury, inflammatory responses, and the development and progression of DAI.

### 4.4. Neuropsychological Assessment

Although neuropsychological assessment as a noninvasive form of diagnosis cannot be used to quantify DAI, it can be used to indirectly show the efficacy of clinical treatment according to the differences in consciousness and cognitive disorders of patients in acute and subacute states. Studies have shown that cognitive disorder is related to the site of injury, correlated in some extent to the state of the white matter connected to specific functional areas. Increasing numbers of investigators have attempted to discern clinical efficacy directly through digitalized neuronal evaluation.

According to the different standards, a variety of partition can be made to the neuropsychological test. The most common ones are divided into a single test and battery of tests. And two common neuropsychological tests are listed as follows.

#### 4.4.1. Halstead-Reitan Neuropsychological Battery (HRB)

The test concludes infants, children, and adults, three versions. And the test is divided into part for verbal test and others for nonverbal test. The revised HRB test battery mainly surveys the following ten aspects: category test, touch operation test, music rhythm test, finger tapping test, Halstead-Wepman aphasia screening test, voice perception test, on one side of the edge test, grip strength test, the attachment test, and perceptual disorder test. Each subtest has different age norm. This set of tests use demarcation points as the norm (the critical points) to distinguish pathology. Then according to the abnormal test counting damage index damage index = abnormal test number/total number. The HRB assessment scale is listed in Table 1.

#### 4.4.2. Luria-Nebraska Neuropsychological Battery, LNNB

LNNB has 1980 and 1985 two versions. The first version includes 269 projects, a total of 11 subtests. The second version added intermediate memory subtest.

There are 11 subtests that constituted the first edition of LNNB and include sports test, rhythm test, touch test, visual test, feeling type words, expressive words, writing test, reading test, math quiz, memory test, and intellectual processes test. And LNNB has three additional scales, as the disease symptoms characteristic scale (qualitative scale), the left hemisphere lateralization of scale, and right side of the scale. These scales are from the previous 11 subtests. Each project of LNNB adopted 3-level scoring mode: “0” is normal, “1” represents borderline state, and “2” indicates exception. Each subtest scores accumulation is LNNB original scores. The more scores shows the heavier damage maybe.

## Figures and Tables

**Table 1:** HRB assessment scale.

Damage index	Pathologic state
0.00–0.14	normal
0.15–0.29	Borderline state
0.30–0.43	Mild brain injury
0.44–0.57	Moderate brain injury
>0.58	Severe brain injury

## Additional Points

DAI occurs when external instant mechanical forces, such as shear force and tension, cause axon swelling and progress to axon breakage. The pathological mechanism of DAI is complicated: axon swelling causes the formation of axonal retraction balls, and calcium influx elicits a series of ion imbalances, impairs the mitochondria, and activates caspase-mediated programmed cell death. Calpain hydrolyzes structural proteins and degrades the cytoskeleton network. Glial cells also take part in the overall process. Pathological cascades take place after DAI. The pathological mechanism is still unclear. Due to the complicated pathological mechanisms underlying DAI, there is no uniform standard for its clinical diagnosis. Currently, most of the commonly used diagnostic standards are noninvasive methods, such as neuropsychological assessment, CT/MRI imaging, and biochemical markers. However, each individual method of diagnosing DAI has its own specific limitations. In the future, after further investigation and assessment of the pathological mechanism underlying DAI, a multimechanism form of diagnosis may be available. Ideally, this method will be more reliable and sensitive and facilitate location of the injured site and range of DAI and realize the increase of clinical efficacy for treatment of DAI.

## References

[1] Levi L., Guilburd J. N., Lemberger A., Soustiel J. F., Feinsod M. (1990). Diffuse axonal injury: analysis of 100 patients with radiological signs. *Neurosurgery*.

[2] Staal J. A., Dickson T. C., Chung R. S., Vickers J. C. (2007). Cyclosporin-a treatment attenuates delayed cytoskeletal alterations and secondary axotomy following mild axonal stretch injury. *Developmental Neurobiology*.

[3] Albensi B. C., Knoblach S. M., Chew B. G. M., O'Reilly M. P., Faden A. I., Pekar J. J. (2000). Diffusion and high resolution MRI of traumatic brain injury in rats: time course and correlation with histology. *Experimental Neurology*.

[4] Reeves T. M., Phillips L. L., Povlishock J. T. (2005). Myelinated and unmyelinated axons of the corpus callosum differ in vulnerability and functional recovery following traumatic brain injury. *Experimental Neurology*.

[5] Huang L., Applegate P. M., Gatling J. W., Mangus D. B., Zhang J., Applegate R. L. (2014). A systematic review of neuroprotective strategies after cardiac arrest: from bench to bedside (part II-comprehensive protection). *Medical Gas Research*.

[6] Thurmond V. A., Hicks R., Gleason T. (2010). Advancing integrated research in psychological health and traumatic brain injury: common data elements. *Archives of Physical Medicine and Rehabilitation*.

[7] Mac Donald C. L., Johnson A. M., Cooper D. (2011). Detection of blast-related traumatic brain injury in U.S. military personnel. *The New England Journal of Medicine*.

[8] McGowan J. C., Yang J. H., Plotkin R. C. (2000). Magnetization transfer imaging in the detection of injury associated with mild head trauma. *American Journal of Neuroradiology*.

[9] Sotelo J. R., Canclini L., Kun A. (2014). Glia to axon RNA transfer. *Developmental Neurobiology*.

[10] Huang L., Obenaus A. (2011). Hyperbaric oxygen therapy for traumatic brain injury. *Medical Gas Research*.

[11] Jeanneret V., Yepes M. (2016). The plasminogen activation system promotes dendritic spine recovery and improvement in neurological function after an ischemic stroke. *Translational Stroke Research*.

[12] Mondello S., Robicsek S. A., Gabrielli A. (2010). *α*II-spectrin breakdown products (SBDPs): diagnosis and outcome in severe traumatic brain injury patients. *Journal of Neurotrauma*.

[13] Merali Z., Leung J., Mikulis D., Silver F., Kassner A. (2015). Longitudinal assessment of imatinib’s effect on the blood-brain barrier after ischemia/reperfusion injury with permeability MRI. *Translational Stroke Research*.

[14] Xiao-Sheng H., Sheng-Yu Y., Xiang Z., Zhou F., Jian-Ning Z., Li-Sun Y. (2000). Diffuse axonal injury due to lateral head rotation in a rat model. *Journal of Neurosurgery*.

[15] Friess S. H., Ichord R. N., Owens K. (2007). Neurobehavioral functional deficits following closed head injury in the neonatal pig. *Experimental Neurology*.

[16] Tate C. M., Wang K. K. W., Eonta S. (2013). Serum brain biomarker level, neurocognitive performance, and self-reported symptom changes in soldiers repeatedly exposed to low-level blast: a breacher pilot study. *Journal of Neurotrauma*.

[17] Badaut J., Bix G. J. (2014). Vascular neural network phenotypic transformation after traumatic injury: potential role in long-term sequelae. *Translational Stroke Research*.

[18] Raghupathi R., Huh J. W. (2007). Diffuse brain injury in the immature rat: evidence for an age-at-injury effect on cognitive function and histopathologic damage. *Journal of Neurotrauma*.

[19] Lee S. R., Choi B., Paul S. (2015). Depressive-like behaviors in a rat model of chronic cerebral hypoperfusion. *Translational Stroke Research*.

[20] Maegele M., Riess P., Sauerland S. (2005). Characterization of a new rat model of experimental combined neurotrauma. *Shock*.

[21] Park E., Liu E., Shek M., Park A., Baker A. J. (2007). Heavy neurofilament accumulation and *α*-spectrin degradation accompany cerebellar white matter functional deficits following forebrain fluid percussion injury. *Experimental Neurology*.

[22] Zhao J., Chen Z., Xi G., Keep R. F., Hua Y. (2014). Deferoxamine attenuates acute hydrocephalus after traumatic brain injury in rats. *Translational Stroke Research*.

[23] Singh A., Lu Y., Chen C., Kallakuri S., Cavanaugh J. M. (2006). A new model of traumatic axonal injury to determine the effects of strain and displacement rates. *Stapp Car Crash Journal*.

[24] Pekny M., Wilhelmsson U., Pekna M. (2014). The dual role of astrocyte activation and reactive gliosis. *Neuroscience Letters*.

[25] Albinsson S., Nordström I., Swärd K., Hellstrand P. (2008). Differential dependence of stretch and shear stress signaling on caveolin-1 in the vascular wall. *American Journal of Physiology—Cell Physiology*.

[26] Ekmark-Lewén S., Flygt J., Kiwanuka O. (2013). Traumatic axonal injury in the mouse is accompanied by a dynamic inflammatory response, astroglial reactivity and complex behavioral changes. *Journal of Neuroinflammation*.

[27] Back S. A., Han B. H., Luo N. L. (2002). Selective vulnerability of late oligodendrocyte progenitors to hypoxia-ischemia. *Journal of Neuroscience*.

[28] Ohta K., Iwai M., Sato K. (2003). Dissociative increase of oligodendrocyte progenitor cells between young and aged rats after transient cerebral ischemia. *Neuroscience Letters*.

[29] Chen H., Xu X., Teng J. (2015). CXCR4 inhibitor attenuates allergen-induced lung inflammation by down-regulating MMP-9 and ERK1/2. *International Journal of Clinical and Experimental Pathology*.

[30] Schwab M. E. (2010). Functions of Nogo proteins and their receptors in the nervous system. *Nature Reviews Neuroscience*.

[31] Schiraldi M., Raucci A., Muñoz L. M. (2012). HMGB1 promotes recruitment of inflammatory cells to damaged tissues by forming a complex with CXCL12 and signaling via CXCR4. *The Journal of Experimental Medicine*.

[32] Polyakova M., Schroeter M. L., Elzinga B. M. (2015). Brain-derived neurotrophic factor and antidepressive effect of electroconvulsive therapy: systematic review and meta-analyses of the preclinical and clinical literature. *PLoS ONE*.

[33] Dallasen R. M., Bowman J. D., Xu Y. (2011). Isoflurane does not cause neuroapoptosis but reduces astroglial processes in young adult mice. *Medical Gas Research*.

[34] Tong K. A., Ashwal S., Holshouser B. A. (2003). Hemorrhagic shearing lesions in children and adolescents with posttraumatic diffuse axonal injury: improved detection and initial results. *Radiology*.

[35] Takayama H., Kobayashi M., Sugishita M., Mihara B. (2000). Diffusion-weighted imaging demonstrates transient cytotoxic edema involving the corpus callosum in a patient with diffuse brain injury. *Clinical Neurology and Neurosurgery*.

[36] Bener A., Omar A. O. K., Ahmad A. E., Al-Mulla F. H., Abdul Rahman Y. S. (2010). The pattern of traumatic brain injuries: a country undergoing rapid development. *Brain Injury*.

[37] Eckermann J. M., Chen W., Jadhav V. (2011). Hydrogen is neuroprotective against surgically induced brain injury. *Medical Gas Research*.

[38] Zhang Y. P. I., Cai J., Shields L. B. E., Liu N., Xu X.-M., Shields C. B. (2014). Traumatic brain injury using mouse models. *Translational Stroke Research*.

[39] Staal J. A., Dickson T. C., Gasperini R., Liu Y., Foa L., Vickers J. C. (2010). Initial calcium release from intracellular stores followed by calcium dysregulation is linked to secondary axotomy following transient axonal stretch injury. *Journal of Neurochemistry*.

[40] Haelewyn B., Chazalviel L., Nicole O., Lecocq M., Risso J.-J., Abraini J. H. (2011). Moderately delayed post-insult treatment with normobaric hyperoxia reduces excitotoxin-induced neuronal degeneration but increases ischemia-induced brain damage. *Medical Gas Research*.

[41] Yang Y., Kimura-Ohba S., Thompson J., Rosenberg G. A. (2016). Rodent models of vascular cognitive impairment. *Translational Stroke Research*.

[42] Lioutas V.-A., Alfaro-Martinez F., Bedoya F., Chung C.-C., Pimentel D. A., Novak V. (2015). Intranasal insulin and insulin-like growth factor 1 as neuroprotectants in acute ischemic stroke. *Translational Stroke Research*.

[43] Mammis A., McIntosh T. K., Maniker A. H. (2009). Erythropoietin as a neuroprotective agent in traumatic brain injury. Review. *Surgical Neurology*.

[44] Kilinc D., Gallo G., Barbee K. A. (2009). Mechanical membrane injury induces axonal beading through localized activation of calpain. *Experimental Neurology*.

[45] Joseph M. J. E., Caliaperumal J., Schlichter L. C. (2016). After intracerebral hemorrhage, oligodendrocyte precursors proliferate and differentiate inside white-matter tracts in the rat striatum. *Translational Stroke Research*.

[46] An C., Jiang X., Pu H. (2016). Severity-dependent long-term spatial learning-memory impairment in a mouse model of traumatic brain injury. *Translational Stroke Research*.

[47] Yokobori S., Mazzeo A. T., Hosein K., Gajavelli S., Dietrich W. D., Bullock M. R. (2013). Preconditioning for traumatic brain injury. *Translational Stroke Research*.

[48] Andriessen T. M. J. C., Jacobs B., Vos P. E. (2010). Clinical characteristics and pathophysiological mechanisms of focal and diffuse traumatic brain injury. *Journal of Cellular and Molecular Medicine*.

